# Curcumin affects function of Hsp90 and drug efflux pump of *Candida albicans*

**DOI:** 10.3389/fcimb.2022.944611

**Published:** 2022-09-27

**Authors:** Yean Sheng Lee, Xinyue Chen, Tria Widiasih Widiyanto, Kanami Orihara, Hiroyuki Shibata, Susumu Kajiwara

**Affiliations:** ^1^ School of Life Science and Technology, Tokyo Institute of Technology, Yokohama, Japan; ^2^ Graduate School of Medicine, Akita University, Akita, Japan

**Keywords:** antifugal activity, Hsp90, CDR1, post-transcripional control, pathogenic fungus

## Abstract

*Candida albicans* is a pathogenic yeast that causes candidiasis in immunocompromised patients. The overuse of antifungal drugs has led to the development of resistance to such drugs by this fungus, which is a major challenge in antifungal chemotherapy. One approach to this problem involves the utilization of new natural products as an alternative source of antifungals. Curcumin, one such natural product, has been widely studied as a drug candidate and is reported to exhibit antifungal activity against *C. albicans*. Although studies of the mechanism of curcumin against human cancer cells have shown that it inhibits heat shock protein 90 (Hsp90), little is known about its function against *C. albicans*. In this paper, using a doxycycline-mediated *HSP90* strain and an *HSP90*-overexpressing strain of *C. albicans*, we demonstrated that the curcumin triggered a decrease in Hsp90 by affecting it at the post-transcriptional level. This also led to the downregulation of *HOG1* and *CDR1*, resulting in a reduction of the stress response and efflux pump activity of *C. albicans*. However, the inhibition of *HSP90* by curcumin was not due to the inhibition of transcription factors *HSF1* or *AHR1*. We also found that curcumin can not only decrease the transcriptional expression of *CDR1*, but also inhibit the efflux pump activity of Cdr1. Hence, we conclude that disruption of *HSP90* by curcumin could impair cell growth, stress responses and efflux pump activity of *C. albicans*.

## Introduction

*Candida albicans* is an opportunistic pathogenic fungus, one of many microorganisms including bacteria and other fungi that normally coexist without causing harm within the human body. An infection caused by this fungus is called candidiasis, and healthy people are protected from candidiasis by both innate and acquired immunities. However, this microorganism can cause both superficial and systemic infections in humans, mainly in immunocompromised individuals such as AIDS patients, neonates, people with debilitating disease, or those who have undergone extensive surgery and are hospitalized for an extended period (Eggimann et al., 2003; Hajjeh et al., 2004; Patterson, 2005; Pfaller and Diekema, 2007; Cisterna et al., 2010; Brown et al., 2012). *C. albicans* has become the most common pathogenic fungi in humans, with the incidence of fungal infections having increased greatly in recent decades. This is due to the growing proportion of the worldwide human population that is immunocompromised and aged. Significant progress has been made in antifungal chemotherapy (Cowen et al., 2002; Carrillo-Munoz et al., 2006; Ortega et al., 2011), but the appearance of antifungal resistant strains and the limits of effective and selective antifungals are major problems in the treatment of clinical fungal infections (Almirante et al., 2005). It is therefore urgent to develop novel antifungal agents that are both safe and effective. One strategy is to combine the search for new potential targets with the screening of promising new antifungal agents (Carrillo-Munoz et al., 2006).

To develop novel antifungals, there have been numerous efforts to identify natural compounds that exhibit effective antifungal activity, and one such compound, curcumin, has been of great interest to the scientific community due to its chemotherapeutic properties (Hatcher et al., 2008). Curcumin is a yellow-pigmented polyphenolic compound that is derived from the roots of the *Curcuma longa*, a plant native to India and other parts of southeast Asia (Limtrakul et al., 2004; Jurenka, 2009; Rowe et al., 2009). In India and China, this compound has been widely used as a cosmetic and sometimes as medicine for the treatment of wounds and inflammation (Govindarajan and Stahl, 1980; Ammon and Wahl, 1991; Strimpakos and Sharma, 2008). Previous studies have found that curcumin has anti-carcinogenic, antioxidant, anti-inflammatory, and antimicrobial properties, as well as exhibiting hypoglycemic effects in humans (Jurenka, 2009). In addition, the safety of curcumin has been studied in animal models and human clinical trials, which have shown that its toxicity is low even when administered at high doses (Bhavani Shankar et al., 1980; Qureshi et al., 1992). Previous studies using both biochemical, genetic or both approaches have shown that the curcumin exhibits antifungal activity against *C. albicans via* oxidative stress, inhibiting hyphal development, disrupting cell wall integrity and plasma membrane, modulates proteolytic enzyme activity, altering the membrane-associated properties of ATPase activity, modulating efflux pumps, synergizing with antifungal azoles, and inhibiting biofilm formation (Sharma et al., 2009; Sharma et al., 2010a; Sharma et al., 2010b; Neelofar et al., 2011; Garcia-Gomes et al., 2012; Kumar et al., 2014; Lee and Lee, 2014; Shahzad et al., 2014; Thakre et al., 2016; Alalwan et al., 2017; Chen et al., 2018; Andrade et al., 2019; Hamzah et al., 2020; Cheraghipour et al., 2021; Dong et al., 2021; Lasrado et al., 2022).

However, little is known about the molecular mechanisms of curcumin’s effect on yeast cells. In human cancer therapeutic, a series of recent studies show that curcumin inhibits heat-shock protein 90 (Hsp90) in human cancer cells (Zhang et al., 2007; Giommarelli et al., 2010; Anand et al., 2012; Khan et al., 2012; Li et al., 2012; Bhullar et al., 2015; Lv et al., 2015; Zheng et al., 2016; Ye et al., 2017; Fan et al., 2018; Forouzanfar et al., 2019; Liu et al., 2020; Surma et al., 2022). Due to the high conservation of Hsp90 across species and the high degree of homology between *C. albicans* and human Hsp90, curcumin is expected to have disruptive effects on Hsp90 in fungal cells as well (Whitesell et al., 2019). But to our knowledge, no study has examined the relationship between fungal *HSP90* and curcumin. To illuminate this uncharted area, we examined the effects of curcumin on the *HSP90* of *C. albicans* by utilizing a defective conditional *HSP90* mutant and an *HSP90*-overexpressing strain. Since the *HSP90* gene is essential for *C. albicans*, a doxycycline (Dox)-regulated expression system was adopted in *C. albicans* by replacing the promoter of *HSP90* with the *tetO* system. This allowed us to control the gene expression of *HSP90* by supplementing Dox, which binds to *tetR* to prevent the transcription of *HSP90*. To better understand the mechanism of curcumin, we also overexpressed *HSP90* by replacing its promoter with the constitutive *ADH1* promoter.

In addition, the low bioavailability and poor stability of curcumin have been highlighted as major problems for therapeutic application (Blasius et al., 2004; Anand et al., 2007; Fang et al., 2013). Therefore, many studies have been carried out attempting to improve the bioavailability and stability of curcumin by modification of the molecular structure. GO-Y030, a curcumin derivative designed by Shibata *et al.* (Shibata et al., 2009), has been reported to possess greater anti-cancer properties than curcumin itself and is significantly less toxic (Gritsko et al., 2006; Cen et al., 2009; Hutzen et al., 2009; Kudo et al., 2011; Mohan Yallapu et al., 2012). However, the antifungal activity of curcumin GO-Y030 has not yet been studied. This study aimed to study the effect of curcumin and its derivative GO-Y030 on Hsp90 of *C. albicans*.

## Materials and methods

### Chemical and antifungal agents

Curcumin (Wako, Japan) stock solutions were prepared in sterile dimethyl sulfoxide (DMSO). The curcumin analogue GO-Y030 was provided by Akita University, Japan. Its stock solution was prepared in sterile DMSO and stored at 4°C. Nile red (Wako, Japan) stock solution was prepared in ethanol and stored at 4°C. Doxycycline (Wako, Japan) was prepared in distilled water and stored at 4°C. Curcumin compounds and all dye solutions were kept in the dark to prevent light exposure.

### Strain and plasmid construction

*C. albicans* strains used in this study are listed in the **Table 1**. All strains were routinely grown in Yeast Extract Peptone Dextrose (YPD; 1% yeast extract, 2% peptone, 2% glucose) or synthetic defined medium (SD; 0.67% yeast nitrogen base without amino acids, 0.079% complete supplement mixture without uracil, 2% glucose) on plates with 2% agar at 37°C. All strains were maintained and stored at 4°C. All strains were stored as frozen stocks with 15% glycerol at −80°C. Construction of the Dox-mediated *HSP90* gene mutant and *HSP90* overexpression mutant were performed as previously described (Reuß and Morschhäuser, 2006; Lai et al., 2016). The plasmids and primers used in this study are listed in the **Tables 2**, **3**.

**Table 1:** Strains used in this study.

Strain	Parental strain	Genotype	Reference
THE1	SC5314	***ade2*::hisG/*ade2*::hisG *ura3*::imm434/*ura3*::imm434** ***ENO1*/*eno1*::*ENO1-tetR-ScHAP4AD-3×HA-ADE2***	(Nakayama et al., 2000)
*HSP90/hsp90Δ*	THE1	***ade2*::hisG/*ade2*::hisG *ura3*::imm434/*ura3*::imm434** ***ENO1*/*eno1*::*ENO1-tetR-ScHAP4AD-3×HA-ADE2*** ***hsp90*::frt/*HSP90***	This study
*tetO-HSP90/hsp90Δ*	*HSP90/hsp90Δ*	***ade2*::hisG/*ade2*::hisG *ura3*::imm434/*ura3*::imm434** ***ENO1*/*eno1*::*ENO1-tetR-ScHAP4AD-3×HA-ADE2*** ***hsp90*::frt/*hsp90*::*URA3-97t-HSP90***	This study
*P_ADH1_*-*HSP90*	THE1	***ade2::hisG/ade2::hisG ura3::imm434/ura3::imm434*** ***ENO1/eno1::ENO1-tetR-ScHAP4AD-3×HA-ADE2*** ***HSP90/hsp90::URA3-ADH1-HSP90***	This study

**Table 2:** Plasmids used in this study.

Name	Reference
pSFS2	(Reuß et al., 2004)
pSFS2-SAT1/2	This study
p97CAU1	(Nakayama et al., 2000)
p97CAU1-HSP1/2	This study
p97CAU1A-HSP1/2	This study

**Table 3:** Oligonucleotide primers used for cloning in this study.

Name	Sequence (5’-3’)	Reference
SAT1.FOR	AAGGTACCGAGGCCCTGAGGAACTTGAC	This study
SAT1.REV	AAGGGCCCACGGGAGGAGTTGATAAACTGG	This study
SAT2.FOR	AAGCGGCCGCACACCAGAAGGGCTACAGTT	This study
SAT2.REV	AAGAGCTCATGACATGACTTGCGTGGGT	This study
HSP1.FOR	AAGGGCCCTGCTCACGGAACCAGAACTT	This study
HSP1.REV	AAATCGATCCAACGGAGACCACTGGAAA	This study
HSP2.FOR	AAACTAGTGTTCATTATGGCTGACGCAAAAG	This study
HSP2.REV	AACCGCGGCAACATGGTACCACGACCCA	This study
ADH1.FOR	AAGGGCCCTGCTCACGGAACCAGAACTT	This study
ADH1.REV	AACCGCGGCAACATGGTACCACGACCCA	This study

Underlined sequence indicates introduced restriction sites.

The *HSP90/hsp90Δ* strain was constructed as follows: one of the chromosomal *HSP90* alleles in the *C. albicans* strain THE1 was deleted using the SAT-flipper method, as described previously (Reuß et al., 2004). SAT1 was amplified with primers SAT1.FOR and SAT1.REV. SAT2 was amplified with primers SAT2.FOR and SAT2.REV. From plasmid pSFS2, pSFS2-SAT1/2 was generated by cloning PCR fragments SAT1 and SAT2 into the respective sites. pSFS2-SAT1/2 was digested with *Kpn*I and *Sac*I to release the disruption cassette and transformed into the TR transactivator gene-containing strain THE1 by electroporation (Thompson et al., 1998). Nourseothricin-resistant transformants were selected on YPD agar plates containing 200 µg/mL of nourseothricin. After the induction of FLP recombinase by growing in YPD medium, nourseothricin-sensitive colonies were selected on the YPD plates containing 25 µg/mL of nourseothricin according to their colony size.

The *tet-HSP90/hsp90Δ* strain was constructed as follows: two regions spanning positions −711 to −138 (HSP1) and positions −7 to +528 (HSP2) relative to the ATG start codon of the *HSP90* open reading frame were PCR amplified using primer pairs HSP1.FOR with HSP1.REV and HSP2.FOR with HSP2.REV. These amplification products were then cloned into the respective sites of plasmid p97CAU1 (Nakayama et al., 2000) to form p97CAU1-HSP1/2. This plasmid was then digested with *Apa*I and *Sac*II to liberate the entire 3 kb promoter-replacing construct and transformed into the *HSP90/hsp90Δ* strain using electroporation (Thompson et al., 1998). Ura+ transformants were selected on SD agar plates without uracil.

The *HSP90*-overexpressing mutant (*P_ADH1_*-*HSP90*) was constructed as follows: the TR promoter of plasmid p97CAU1-HSP1/2 was replaced by the *ADH1* promoter to form plasmid p97CAU1A-HSP1/2. Briefly, the *ADH1* promoter was amplified by ADH1.FOR and ADH1.REV using *C. albicans* SC5314. Then, the *ADH1* promoter fragment replaced the TR promoter of plasmid p97CAU1-HSP1/2 at restriction sites *Spe*I and *Sma*I to form plasmid p97CAU1A-HSP1/2. Similar to the construction of the *tet-HSP90/hsp90Δ* strain, this plasmid was then digested with *Apa*I and *Sac*II to liberate the entire 2.505 kb promoter-replacing construct and transformed into *C. albicans* THE1 using electroporation (Thompson et al., 1998). Ura+ transformants were selected on SD agar plates without uracil.

### *In vitro* susceptibility test

Minimum inhibitory concentration (MIC) assays were carried out in flat-bottom, 96-well microtiter plates (Iwaki, Japan) using serial broth microdilution with minor modifications (Rodríguez-Tudela et al., 2003). The MIC_80_ was defined as the concentration of the antifungal compound that inhibits 80% of the growth of cells as compared with the control. MIC tests were set up in a final volume of 200 µL per well with 2-fold serial dilutions of curcumin compounds in YPD. Gradients of curcumin compounds were diluted from 250 µg/mL down to 0 µg/mL. *C. albicans* strains SC5314 or *P_ADH1_-HSP90* were grown in YPD overnight at 37°C. Then the cells were collected and washed by phosphate-buffered saline (PBS) three times. The number of cells was adjusted to 1 × 10^3^ cells/mL in YPD. 100 µL of each strain was inoculated into each well. The plates were then incubated at 37°C for 24 h. The endpoint of the MIC assay was determined by Varioskan Lux (Thermo Scientific, Japan) at an absorbance of 530 nm and corrected for background from the corresponding medium. Each strain was tested in triplicate for each curcumin compound. The optical densities were averaged for triplicate measurements.

### qRT-PCR analysis

To elucidate the potential mechanism by which curcumin inhibits the growth of *C. albicans*, *HSP90*, *HSF1*, *AHR1*, *HOG1*, *CDR1*, *CDR2*, and *MDR1* gene expression analysis was performed using qRT-PCR. Briefly, the wildtype, *tet-HSP90/hsp90Δ*, or *P_ADH1_-HSP90* strains were cultured at 37°C in YPD until they reached the exponential phase. The cells were then collected, washed, and resuspended in YPD containing curcumin compounds or Dox. A drug-free control was included for each experiment. The samples were then pelleted and washed with Diethyl pyrocarbonate (DEPC)-treated water. The pellet was used to extract the RNA using hot acidic phenol and purified using ethanol (Collart and Oliviero, 1993). The purity and concentration of the extracted RNA were verified using GeneQuant 100 (Biochrom, Japan). RNA was then converted to cDNA using ReverTra Ace™ qRT RT Master Mix with gDNA Remover (Toyobo, Japan) following the manufacturer’s recommended protocol. Gene expression was analyzed by qRT-PCR using the StepOne™ Real-Time PCR System (Thermo Fisher, Japan). The *ACT1* housekeeping gene was used as a reference, and the relative gene expression (fold change) was determined by the 2^-ΔΔCT^ method (Livak and Schmittgen, 2001). The primers used for qRT-PCR are listed in **Table 4**.

**Table 4:** Oligonucleotide primers used for qRT-PCR in this study.

Name		Sequence (5’ to 3’)
*ACT1* primers	Forward	GTCTTTGTACTCTTCTGGTAGAACCACCGG
	Reverse	GGACAAATGGTTGGTCAAGTCTCTACCAGC
*HSP90* primers	Forward	TGCTCCAGCTGCCATTAGAACTGG
	Reverse	GGTCTTGTCTTCAGCTCCATCGGTTT
*HSF1* primers	Forward	TCCAACACCTACCCTGGAAC
	Reverse	TGGCAACACTAATGGATGGA
*AHR1* primers	Forward	GGTTGCGTTACCTGTCGAGA
	Reverse	GCAACAGCAGCAACAACAAC
*HOG1* primers	Forward	GTTGAACCGGAGGCTATTGA
	Reverse	TGCCACACCAACAGTTTGAT
*CDR1* primers	Forward	CATGGTCAAGCCATTTTGTG
	Reverse	ATCCATTCTGCTGGATTTGC
*CDR2* primers	Forward	CATGGTCAAGCCATTTTGTG
	Reverse	ATCCATTCTGCTGGATTTGC
*MDR1* primers	Forward	CAAATTCCCACTGCTTTGGT
	Reverse	ACAAACAGCACCCAAACTCC
18s rRNA primers	Forward	GCCAGCGAGTATTAACCTTG
	Reverse	AGGCCTCACTAAGCCATTCA

### Checkerboard assay

The drug combinations were studied by means of a two-dimensional broth microdilution checkerboard procedure using two-antifungal agents as described in the Clinical Microbiology Procedures Handbook (Isenberg, 1992). The checkerboard assays were carried out in flat-bottom, 96-well microtiter plates (Iwaki, Japan) using a serial broth microdilution protocol with minor modifications (Chaturvedi et al., 2008). Tests were set up in a final volume of 200 µL per well with 2-fold serial dilutions of curcumin in YPD medium. Gradients of curcumin and Dox were diluted from 250 µg/mL down to 0.24 µg/mL and from 0.1 µg/mL to 0.00156 µg/mL, respectively. *C. albicans* SC5314 and *tet-HSP90/hsp90Δ* strains were grown in YPD overnight at 37°C. Then the cells were collected and washed by PBS three times, and the number of cells was adjusted to 1 × 10^3^ cells/mL in YPD. 100 µL of each strain was inoculated into each well, and the plates were incubated at 37°C for 24 h. The endpoint of the MIC was determined by Varioskan Lux (Thermo Scientific, Japan) at an absorbance of 530 nm and corrected for background from the corresponding medium. The data were quantitatively displayed with color using the program Java TreeView 1.2.0.

### Stress response

The heat shock and osmotic stress experiments were performed according to the protocol described previously (Enjalbert et al., 2003). Briefly, cells from an overnight culture were transferred to fresh YPD medium containing curcumin compounds and allowed to grow at 37°C for 2 h. A drug-free control was included for each experiment. For heat shock experiments, the cells were then transferred to 42°C. 100 µL aliquots of the control and stress samples were removed at 0 and 20 min. The cells were then diluted and spread on YPD agar plates. For osmotic stress response, the cells were diluted and spread on YPD plates with or without 1 M NaCl. The plates were then incubated at 37°C for 24 h to observe cell growth. Each experiment was repeated three times.

### Nile red accumulation assay and flow cytometry

The Nile red efflux assay was performed according to a previous protocol (Ivnitski-Steele et al., 2009; Keniya et al., 2015; Eldesouky et al., 2018). To determine the relationship between efflux pump activity and curcumin without depleting the *HSP90* gene, curcumin and Nile red were supplemented simultaneously. Briefly, exponential phase wildtype strain was harvested, washed twice with PBS, and resuspended in PBS containing 2% glucose. The cells were then incubated with 125 µg/mL of curcumin and 7 µM of Nile red for 10 min. To determine the relationship between efflux pump activity and depletion of the *HSP90* gene by curcumin, exponential phase wildtype or *P_ADH1_-HSP90* strain were incubated with 125 µg/mL of curcumin for 2 h. Then the cells were washed with PBS, resuspended in PBS containing 2% glucose, and incubated with 7 µM Nile red for 10 min at 37°C. For the *tet-HSP90/hsp90Δ* mutant, cells were incubated with 0.1 µg/mL of Dox for 2 h before treatment with Nile red. For flow cytometry analysis, the accumulation of Nile red was measured using an EC800 (Sony, Japan) flow cytometer with an excitation wavelength of 488 nm and emission filter of 585/42 nm. At least 10,000 events were analyzed in each experiment.

## Results

### Minimum inhibitory concentration of curcumin compounds against *C. albicans*

**Figure 1:**
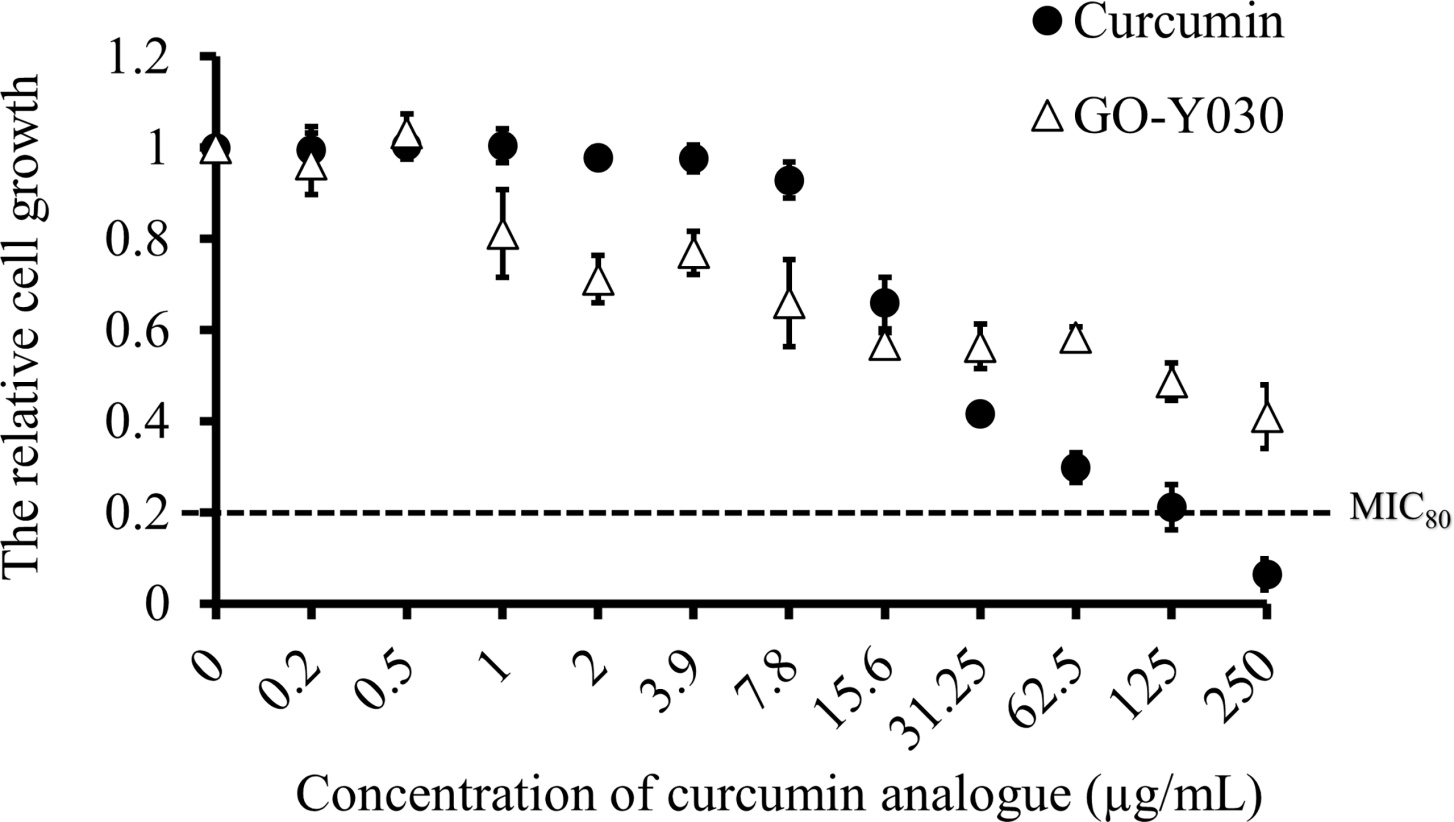
Curcumin compounds exhibit antifungal activity on *C. albicans*. Susceptibility assays of each curcumin analogue were conducted in YPD medium. Growth was measured by absorbance at 530 nm after 24-h incubation at 37°C. Optical densities were averaged for triplicate measurements and normalized relative to no antifungal compound control. Error bars represent standard deviation from the mean of triplicate measurements.

To determine the *in vitro* susceptibility of *C. albicans* SC5314 to curcumin and GO-Y030, broth microdilution assays were performed in YPD medium. Cell growth was determined by absorbance at 530 nm after a 24-h drug treatment. As the concentration of curcumin compounds increased, the cell growth decreased (**Figure 1**). We found that both curcumin compounds inhibited the growth of *C. albicans* dose-dependently. The data also revealed that *C. albicans* was more susceptible to curcumin than to GO-Y030. At a concentration of 250 µg/mL, curcumin inhibited about 80% of cell growth compared to the control (MIC_80_ = 250 µg/mL). In contrast, GO-Y030 inhibited only about 60% of cell growth.

### Curcumin compounds induced depletion of *HSP90* gene expression

**Figure 2:**
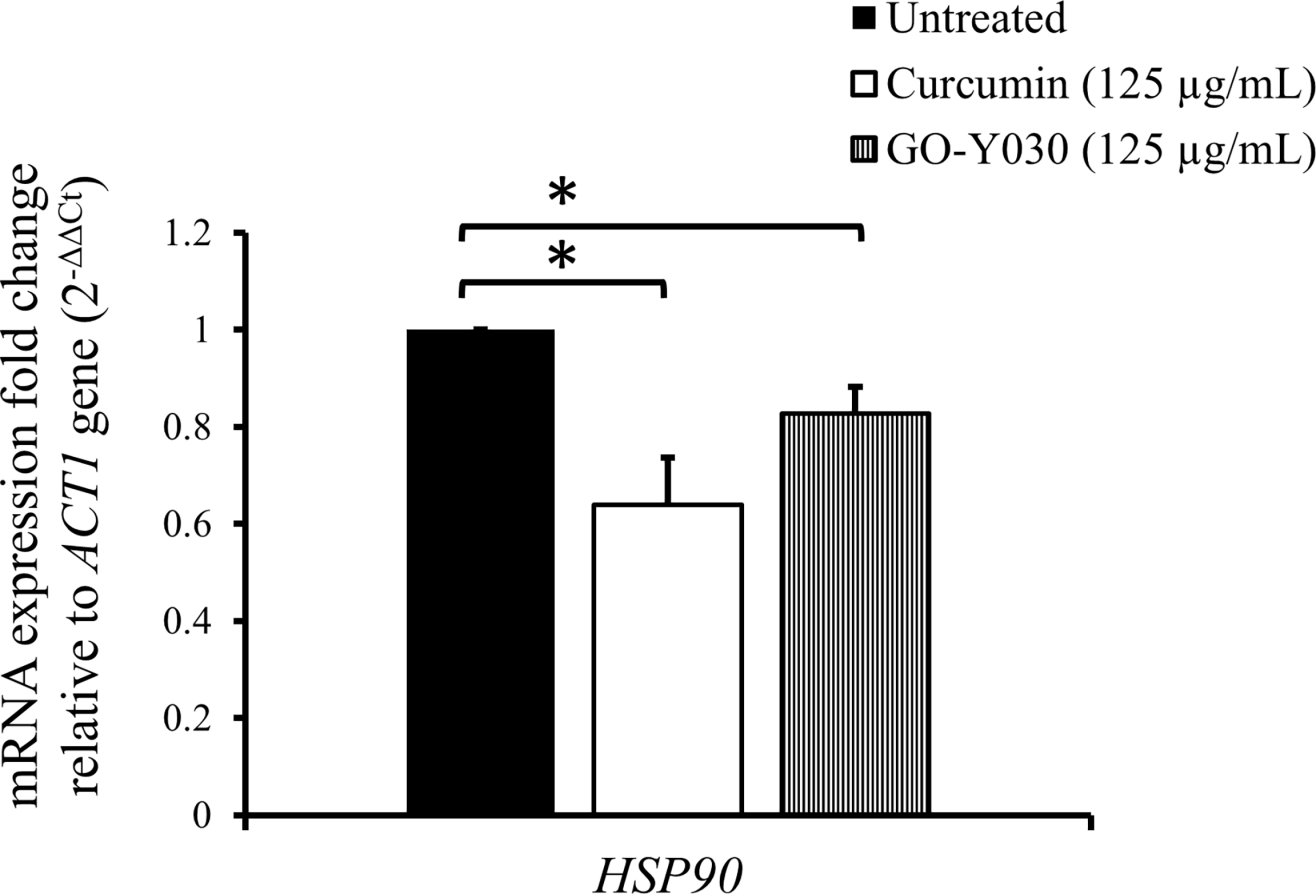
Effect of curcumin compounds on *HSP90* gene expression in *C. albicans* SC5314. Error bars represent standard deviation from the mean of duplicate measurements. * indicates a statistically significant difference (p<0.05) compared to the respective control group.

To investigate the effect of curcumin compounds on the gene expression of *HSP90*, we determined the transcript levels of *HSP90* after mid-log phase *C. albicans* was treated with these compounds for 2 h in YPD at a sub-MIC_80_ concentration (125 µg/mL). **Figure 2** shows the relative transcript levels of *HSP90* in *C. albicans* SC5314 after treatment with the two curcumin compounds. *HSP90* levels in cells with either compound was significantly lower than those in untreated cells. This suggests that both curcumin compounds downregulated *HSP90* expression. Our results also showed that curcumin strongly reduced the transcript levels of *HSP90* compared to GO-Y030. This might explain why curcumin exhibited greater antifungal activity than GO-Y030. These results suggest that the effect of curcumin might be attributable to the inhibition of *HSP90* expression.

### Genetic depletion of *HSP90* in *C. albicans* enhanced susceptibility to curcumin compounds

**Figure 3:**
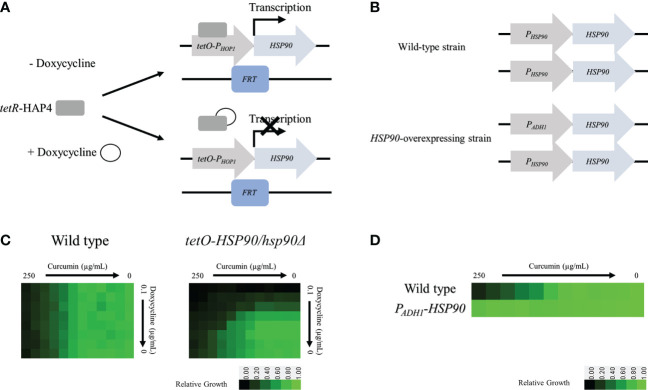
Depletion of *HSP90* increases the sensitivity of cells to curcumin. **(A)** Diagram showing control of the tetR-regulated *tetO-HSP90* fusion gene. In the absence of Dox, tetR can bind to and induce *HSP90* expression. **(B)** Checkboard assays for curcumin in combination with Dox tested against *C albicans* in YPD medium. The optical density of cells at 530 nm (OD_530_) is colored between dark green (no yeast growth) and light green (maximum growth) relative to the control. Data are quantitatively displayed with color using Java TreeView 1.2.0. **(C)** Diagram showing the *HSP90* promoter replaced with the *ADH1* promoter. **(D)** Diagram of the microbroth dilution assay for curcumin against the overexpression mutant *P_ADH1_-HSP90* in YPD medium. The cells were treated with curcumin at 37°C for 24 h.

To determine whether the *HSP90* gene is involved in the function of curcumin, we constructed a conditional *HSP90* mutant and an *HSP90*-overexpressing strain of *C. albicans*. To construct a Dox-repressible allele of *HSP90*, by using the *HSP90/hsp90Δ* strain derived from SC5314, we replaced the *C. albicans HSP90* promoter on the other allele with a *tetO* element to obtain the *tetO-HSP90/hsp90Δ* strain. This strain reduces *HSP90* expression in a Dox-dependent manner (**Figure 3A**). In addition, an *HSP90*-overexpressing strain, *P_ADH1_-HSP90*, was produced by replacing the promoter of *HSP90* with the *ADH1* promoter as shown in **Figure 3B**.

The levels of curcumin sensitivity of the wildtype, *tetO-HSP90/hsp90Δ*, and *P_ADH1_*-*HSP90* strains were compared. For the wildtype, 250 µg/mL of curcumin (MIC_80_) inhibited cell growth completely (**Figure 3C**). This data also revealed that Dox did not affect the curcumin sensitivity of the wild type. For the *tetO-HSP90/hsp90Δ* strain, the MIC_80_ of curcumin was decreased under a high concentration of Dox. At 0.025 µg/mL and 0.05 µg/mL of Dox, the MIC_80_ of curcumin were 15.6 µg/mL and 7.8 µg/mL, respectively. This result showed that the depletion of *HSP90* enhanced sensitivity to curcumin. Hence, the *HSP90* gene might play an important role in resistance to curcumin in *C. albicans*. Moreover, microbroth dilution assays using the *HSP90* overexpression strain (*P_ADH1_-HSP90*) were performed. **Figure 3D** shows the growth of SC5314 and *P_ADH1_-HSP90* after treatment with curcumin. Although growth of the wildtype strain was decreased at a high concentration of curcumin, this compound had no effect on the growth of *P_ADH1_-HSP90* cells. This result indicated that overexpression of the *HSP90* gene suppressed sensitivity to curcumin. Overall, these results suggest that *HSP90* is the key factor in the growth inhibition of *C. albicans* by curcumin.

### Curcumin impaired the stress response of *C. albicans* SC5314

**Figure 4:**
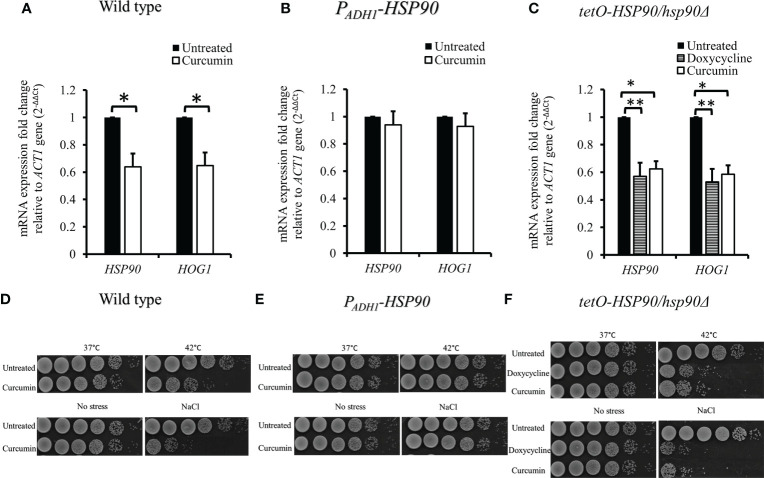
Reduction of *HSP90* gene expression impaired the stress response of *C. albicans*. **(A–C)** Effect of curcumin on *HSP90* and *HOG1* gene expression in the wildtype, *tetO-HSP90/hsp90Δ*, and *P_ADH1_-HSP90* strains after treatment with curcumin. Error bars represent standard deviation from the mean of duplicate measurements. * and ** indicate statistically significant differences (p<0.05 and p<0.01) compared to the respective control group. **(D–F)** For the short-term heat shock stress response, YPD-overnight cultures of the wild type and *P_ADH1_-HSP90* mutant, with or without pre-exposure to curcumin, as well as YPD-overnight cultures of the *tetO-HSP90/hsp90Δ* mutant, with or without pre-exposure to Dox, were serially diluted from 10^6^ to 10^1^ cells (left to right) after exposure to heat shock (42°C for 30 min) or not (control), plated on YPD, and incubated for 24 h at 37°C. For the osmotic stress response, YPD-overnight cultures of the wild type and *P_ADH1_-HSP90* mutant, with or without pre-exposure to curcumin, as well as YPD-overnight cultures of the *tetO-HSP90/hsp90Δ* mutant, with or without pre-exposure to Dox, were serially diluted from 10^6^ to 10^1^ cells (left to right) on YPD plates with or without1 M NaCl. The plates were incubated for 24 h at 37°C.

We then investigated the effect of curcumin on *HOG1* expression as well as that of *HSP90*. **Figure 4A** shows that curcumin reduced the transcript levels of both *HOG1* and *HSP90*. This reduction was restored by the overexpression of *HSP90* in the presence of curcumin (**Figure 4B**). To confirm the relationship between the *HSP90* and *HOG1*, the mRNA levels of *tetO-HSP90/Δhsp90* strain were also analyzed. When *HSP90* expression was suppressed by Dox, the gene expression of *HOG1* was decreased (**Figure 4C**), confirming that the reduction of *HSP90* transcripts led to the reduction of *HOG1* gene expression in *C. albicans*. To further validate the stress responses of the wild type, *P_ADH1_-HSP90*, and *tetO-HSP90/hsp90Δ*, the growth of these strains under thermal and osmotic stresses were analyzed. Stress response tests were performed with heat shock at 42°C for 30 min and with 1 M NaCl. Curcumin reduced the thermotolerance of the wildtype strain after it was exposed to 42°C for 30 min compared to the control (37°C) (**Figure 4D**). In contrast to the wild type, *HSP90* overexpression in *C. albicans* resulted in the growth of cells at 42°C (**Figure 4E**). In addition, the repression of *HSP90* led to a reduction in the tolerance of cells at high temperature (**Figure 4F**). Similarly, curcumin also reduced the osmotic tolerance of *C. albicans* to exposure to 1 M NaCl (**Figure 4D**). Contrastingly, the overexpression of *HSP90* in *C. albicans* retained the tolerance when cells were treated with curcumin (**Figure 4E**). Repression of *HSP90* decreased the osmotic tolerance to a high concentration of NaCl (**Figure 4F**). These results implied that the downregulation of *HSP90* by curcumin impaired the thermal and osmotic stress responses of *C. albicans*.

### Curcumin regulated post-transcriptional processing of *HSP90*

**Figure 5:**
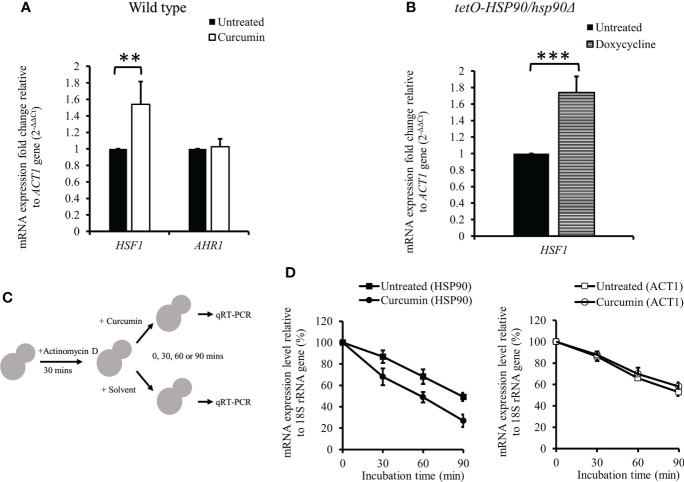
Effect of curcumin on the gene expression of *HSF1* and *AHR1* and the post-transcriptional level of *HSP90*. **(A, B)** Effect of curcumin on *HSF1* and *AHR1* gene expression in the wildtype and *tetO-HSP90/hsp90Δ* strains after treatment with curcumin. Error bars represent standard deviation from the mean of duplicate measurements. ** and *** indicate a statistically significant difference (p<0.01 and p<0.001) compared to the respective control group. **(C)** Diagram showing the post-transcriptional regulation test. Cells were pre-treated with 10 µg/mL of actinomycin D for 30 min, then incubated with curcumin at sub-MIC_80_ for 0, 30, 60, or 90 min. **(D)** Effect of curcumin on *HSP90* and *ACT1* gene expression for the wildtype strain after treatment with actinomycin D and curcumin. Error bars represent standard deviation from the mean of triplicate measurements.

The mRNA levels of transcription factors *HSF1* and *AHR1* were analyzed in the presence of curcumin. Unexpectedly, curcumin initiated the transcriptional induction of *HSF1*, while it did not affect the mRNA of *AHR1* (**Figure 5A**). This suggests that the reduction of the *HSP90* mRNA level by curcumin was not due to the inhibition of *HSF1* transcriptional expression. We confirmed this using the *tetO-HSP90/Δhsp90* mutant. In the presence of the Dox, which suppressed the gene expression of *HSP90*, the *HSF1* transcript level was increased (**Figure 5B**). Therefore, *HSP90* reduction by curcumin was not a result of, but rather led to, the increase of *HSF1* mRNA in *C. albicans*.

We initially thought that Dox-controlled *HSP90* in the *tetO-HSP90/Δhsp90* strain would not be affected by curcumin. However, our results showed that curcumin downregulated *HSP90* in *tetO-HSP90/Δhsp90* as well as in the wild type. We speculated that this might be due to the post-transcriptional regulation of *HSP90* by curcumin. To test this, the mRNA level of *HSP90* was measured after cells were treated with actinomycin D (ActD), an RNA polymerase inhibitor, as shown in **Figure 5C**. The cells were then treated with curcumin for 0, 30, 60, or 90 min. After treatment with ActD, *HSP90* mRNA of the cells degraded gradually, and in addition, it degraded faster in the presence of curcumin than in the control (**Figure 5D**). In contrast, the degradation of *ACT1* mRNA was not affected by the addition of curcumin (**Figure 5D**). This showed that curcumin accelerated the degradation of *HSP90* mRNA specifically and suggests that curcumin induces the post-transcriptional degradation of *HSP90*.

### Curcumin reduced the transcriptional level of *CDR1* by depleting *HSP90*

**Figure 6:**
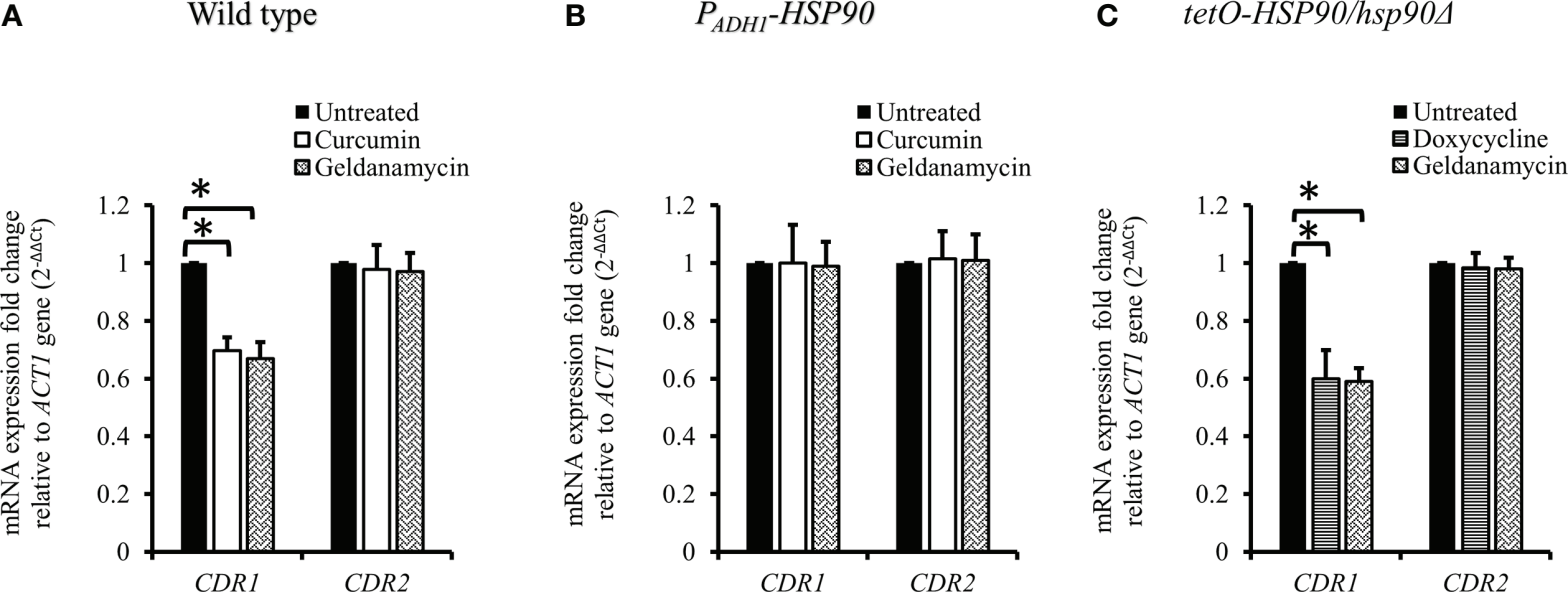
Transcriptional levels of *CDR1* and *CDR2* in the presence of curcumin and geldanamycin. Effects of curcumin on *CDR1* and *CDR2* gene expression for wildtype **(A)**, *P_ADH1_-HSP90* **(B)**, and *tetO-HSP90/hsp90Δ* strains **(C)** after treatment with curcumin or geldanamycin. Error bars represent standard deviation from the mean of duplicate measurements. * indicates a statistically significant difference (p<0.05) compared to the respective control group.

Since curcumin downregulated the transcriptional level of *HSP90*, we expected that curcumin might influence the gene expression of *CDR1*. Therefore, we investigated *CDR1* and *CDR2* (another ABC transporter gene) expression in the presence of curcumin or geldanamycin, which is a known Hsp90 inhibitor. **Figure 6A** shows that *CDR1* gene expression was reduced significantly by curcumin and geldanamycin, but that of *CDR2* was not. A reduction of the *CDRs* by curcumin and geldanamycin did not occur in the *P_ADH1_*-*HSP90* strain (**Figure 6B**). To further confirm that the *CDR1* reduction was due to a reduction of *HSP90* by curcumin and geldanamycin, the effect of depleting *HSP90* on *CDR1* was determined using the *tetO-HSP90/Δhsp90* strain (**Figure 6C**). In the presence of Dox, *CDR1* transcripts decreased while those of *CDR2* remained unchanged. These results suggest that curcumin only downregulated *CDR1* expression *via* a reduction of *HSP90* in *C. albicans*.

### Curcumin reduced efflux pump activity in *C. albicans*

**Figure 7:**
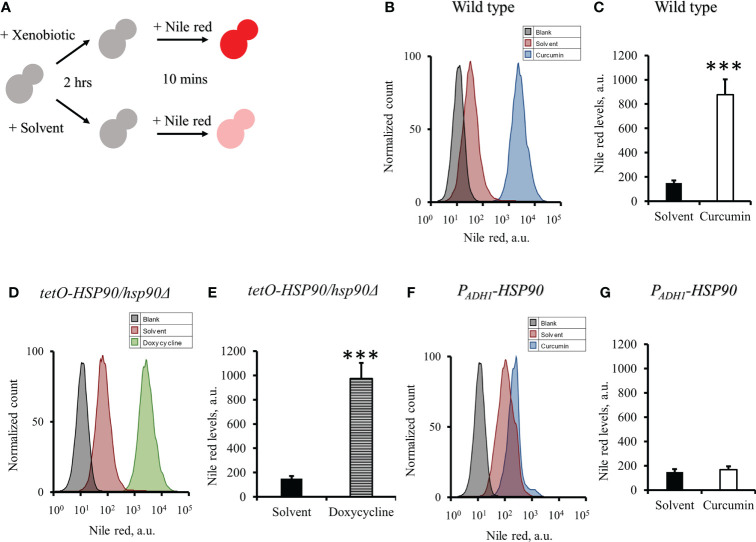
Effect of curcumin on the efflux pump activity of *C. albicans*. **(A)** Diagram showing the Nile red accumulation assay to determine the disruption of efflux pump activity by curcumin. The amounts of Nile red accumulated in *C. albicans* after treatment with curcumin for 2 h were determined for SC5314 **(B, C)** and the *P_ADH1_-HSP90* mutant **(F, G)**, and that in the *tetO-HSP90/hsp90Δ* mutant was determined after treatment with Dox for 2 h **(D, E)** Flow cytometry histograms for mean Nile red fluorescence are shown in **C, E, G**. Error bars represent standard deviation from the mean of duplicate measurements. *** indicates a statistically significant difference (p<0.001) compared to the respective control group.

**Figure 8:**
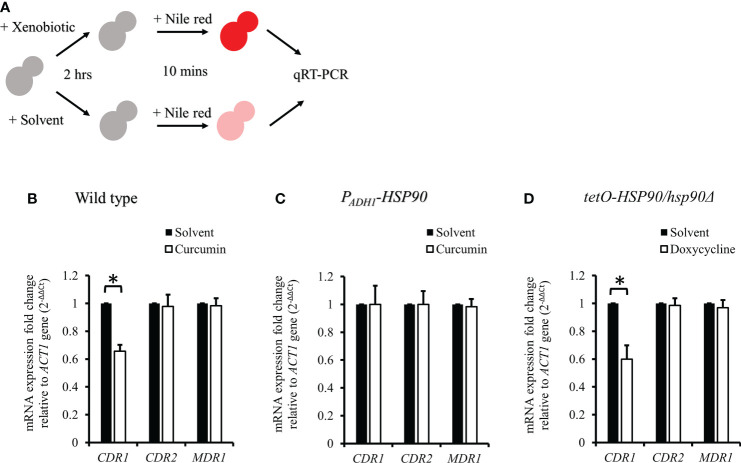
*CDR1*, *CDR2*, and *MDR1* gene expressions in *C. albicans* after treatment with xenobiotics for 2 h and Nile red for 10 min. **(A)** Diagram showing the qRT-PCR experiment. **(B–D)** mRNA amounts of *CDR1*, *CDR2*, and *MDR1* were determined by qRT-PCR in wildtype, *P_ADH1_-HSP90*, and *tetO-HSP90/hsp90Δ* strains. * indicates a statistically significant difference (p<0.05) compared to the respective control group.

As the gene expression of *CDR1* in *C. albicans* was reduced by curcumin, we assumed that the efflux pump activity of the cells might be affected by curcumin. To test this, the Nile red accumulation assay was performed (**Figure 7A**). For the wildtype strain, the addition of curcumin led to a high accumulation of Nile red compared to the control (**Figures 7B, C**). This accumulation was thought to be due to the repression of the *CDR1* gene by the reduction of *HSP90* (**Figure 8**). The relationship between the expression of *HSP90* and the export of Nile red was confirmed by using the *tetO-HSP90/hsp90Δ* strain (**Figures 7D, E**). The overexpression of *HSP90* reversed the accumulation of Nile red by curcumin (**Figures 7F, G**), suggesting that overexpression of the *HSP90* gene maintained *CDR1* expression and restored Nile red extrusion. These results implied that curcumin repressed *HSP90* mRNA resulted in repression of *CDR1* which caused the disruption of efflux pump activity.

### Curcumin also acted as an efflux pump inhibitor

**Figure 9:**
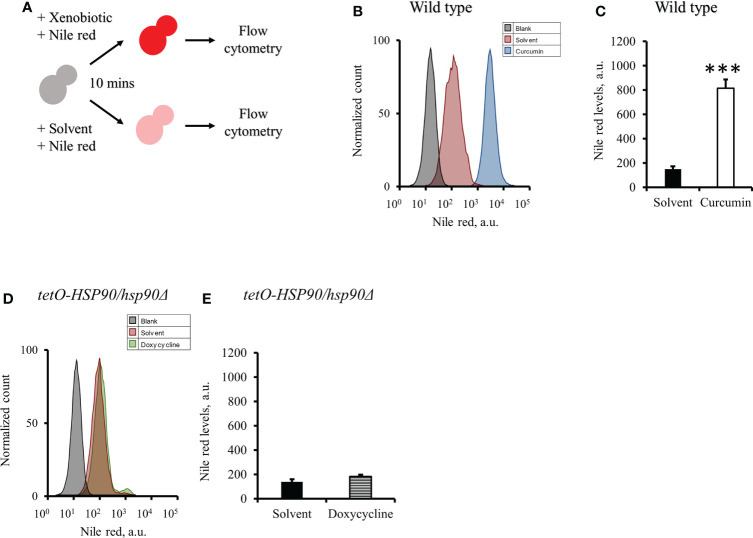
Effect of curcumin on the efflux pump activity of *C. albicans*. **(A)** Diagram showing the Nile red accumulation assay for determining the ability of curcumin to inhibit the efflux pump. **(B)** The amount of the Nile red accumulated in *C. albicans* SC5314 was determined after it was treated with curcumin for 10 minutes. **(C)** The flow cytometry histograms for the mean Nile red fluorescence. **(D)** The amount of the Nile red accumulated in *tetO-HSP90/hsp90Δ* was determined after it was treated with doxycycline for 10 minutes. **(E)** The flow cytometry histograms for the mean Nile red fluorescence. Error bars represented standard deviation from the mean of duplicate measurements. *** indicates a statistically significant difference (p<0.001) compared to the respective control group.

**Figure 10:**
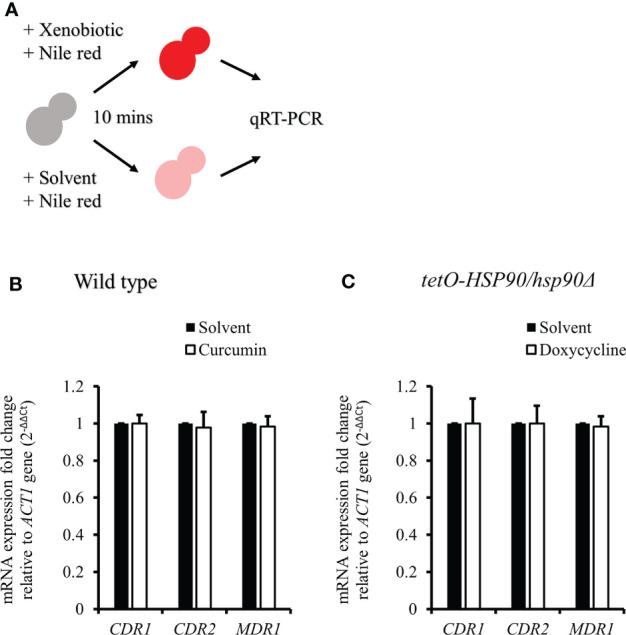
*CDR1*, *CDR2*, and *MDR1* gene expressions in *C. albicans* after treatment with xenobiotics and Nile red for 10 min. **(A)** Diagram showing the qRT-PCR experiment. **(B, C)** The mRNA amounts of *CDR1*, *CDR2*, and *MDR1* were determined by qRT-PCR in wildtype and *tetO-HSP90/hsp90Δ* strains.

To test the effect of curcumin as an efflux pump inhibitor, cells were exposed to curcumin and Nile red simultaneously for 10 min (**Figure 9A**). Based on the flow cytometry results, the addition of curcumin led to the accumulation of Nile red compared to the control (**Figures 9B, C**). However, Nile red did not accumulate in the *tetO-HSP90/hsp90Δ* strain with Dox (**Figures 9D, E**). Since the 10-min pretreatment with curcumin would not alter the gene expression of *HSP90* and *CDR1*, Cdr1 was not decreased (**Figure 10**). These results suggest that, without affecting gene expression of ABC-transporters, curcumin also acts on Cdr1 directly to inhibit its function but Dox does not.

## Discussion

Curcumin, a curcuminoid produced from the rhizomes of *Curcuma longa* (a tiny perennial herb native to India), has been reported to possess anti-inflammatory, anticarcinogenic, and anti-infectious activities (Ravindran et al., 2009; Neelofar et al., 2011). Several shortcomings of curcumin, such as its low bioavailability and poor stability, have been highlighted as major problems in its therapeutic application (Blasius et al., 2004; Anand et al., 2007; Fang et al., 2013). Therefore, many studies have tried to improve the bioavailability and stability of curcumin by structural modification. GO-Y030 reportedly has greater anti-carcinogenic activity and lower toxicity than curcumin (Gritsko et al., 2006; Cen et al., 2009; Hutzen et al., 2009; Kudo et al., 2011; Mohan Yallapu et al., 2012). As the antifungal activity of GO-Y030 has remained unclear, we assessed its inhibitory effect on *C. albicans* growth. In this study, broth microdilution assays demonstrated that curcumin and GO-Y030 had antifungal inhibitory activity against the growth of *C. albicans* SC5314 (**Figure 1**). This result is consistent with previous studies (Andrade et al., 2019; Narayanan et al., 2020) wherein curcumin inhibited the growth of *Candida* strains in a range between 100 µg/mL and 250 µg/mL. Although GO-Y030 had lower antifungal activity than curcumin in this study, to our knowledge, this is the first report showing that GO-Y030 inhibits the growth of *C. albicans*.

Despite the numerous cytotoxic effects of curcumin on *C. albicans* that have been already reported (Sharma et al., 2009; Sharma et al., 2010a; Sharma et al., 2010b; Neelofar et al., 2011; Shahzad et al., 2014; Alalwan et al., 2017; Andrade et al., 2019; Hamzah et al., 2020), the mechanism of this function of curcumin remains unknown. In cancer therapeutics for humans, curcumin has also been reported to be an antitumor compound. This compound influences the *HSP90* gene and its gene product in human tumor cells (Zhang et al., 2007; Giommarelli et al., 2010; Anand et al., 2012; Khan et al., 2012; Li et al., 2012; Liu et al., 2014; Bhullar et al., 2015; Lv et al., 2015; Zheng et al., 2016; Ye et al., 2017; Fan et al., 2018; Forouzanfar et al., 2019). Recent studies have shown that curcumin inhibits ATPase activity in Hsp90 of human cancer cells. The expression of *HSP90* is higher in tumors compared with normal tissues and is important in the maintenance of the stability, integrity, and function of oncogenic proteins. Curcumin and several curcumin derivatives such as C1206, C0818, and CUR3d, have been shown to inhibit Hsp90 function. This results in the dissociation of complexes with client proteins that are important in cell proliferation, cytotoxic damage survivability, and apoptosis, among other functions (Jung et al., 2007; Giommarelli et al., 2010; Lee and Chung, 2010; Bhullar et al., 2015; Fan et al., 2017; Fan et al., 2018). In addition, curcumin has also been found to downregulate *HSP90* gene expression in human cells such as chronic myeloid leukemia cells and human embryonic lung fibroblast cells (Zhang et al., 2007; Lv et al., 2015; Zheng et al., 2016; Ye et al., 2017; Sang et al., 2018). *C. albicans* Hsp90 has also been studied as a heat shock protein that is essential for maintaining homeostasis by promoting the proper folding of abundant client proteins. According to numerous studies, Hsp90 is involved in thermal stability, morphogenesis, cell cycle control, apoptosis, and drug resistance in *C. albicans* (Leach et al., 2012b; O’Meara and Cowen, 2014). Hence, interfering with the physiological activity of Hsp90 could be a promising strategy for treating candidiasis. However, there have been no reports about the effects of curcumin on Hsp90 in this pathogenic fungus. Hsp90 is common among many species and has a conserved amino acid sequence between *C. albicans* and humans (Swoboda et al., 1995), so we expected curcumin to affect Hsp90 in *C. albicans* as well. This study showed that the exposure of *C. albicans* to curcumin or GO-Y030 triggered the transcriptional reduction of *HSP90* (**Figure 2**). This is an important finding in understanding the function of curcumin in *C. albicans*. Unexpectedly, curcumin exhibited greater antifungal activity than GO-Y030 on *C. albicans*, which is the opposite of findings in human tumor cells.

To alter *HSP90* expression in *C. albicans*, we utilized a Dox-mediated *HSP90* strain and an *HSP90*-overexpressing strain to investigate the effects of curcumin on *C. albicans*. Our data revealed that the depletion of *HSP90* in the *tetO-HSP90/hsp90Δ* strain increased susceptibility to curcumin dose-dependently with Dox (**Figure 3C**), and a synergic effect of curcumin and Dox appeared. In contrast, the effect of curcumin disappeared by the overexpression of *HSP90* in the *P_ADH1_-HSP90* strain (**Figure 3D**). These findings indicated that curcumin inhibits the growth of *C. albicans* by repressing *HSP90* function.

Previous reports (Diezmann et al., 2012; Leach et al., 2012a; Leach et al., 2012b; O’Meara and Cowen, 2014) have shown that *HSP90* plays an important role in the survivability of yeast species at high temperatures and high osmotic pressures. The depletion of *HSP90* reduced the thermotolerance of *C. albicans* and led to the decrease of a mitogen-activated protein kinase, Hog1, which plays an important role in the osmotic stress response. This study showed that the depletion of *HSP90* by curcumin resulted in a reduction of *HOG1* (**Figure 4**), suggesting that curcumin also decreased *HOG1* expression and impaired the stress response of *C. albicans.*

In *C. albicans*, protein kinase CK2 and transcription factor Ahr1 operate upstream of Hsp90 to promote cell growth in many environments. In addition, *HSP90* expression is also controlled by the transcription factor Hsf1, whose activation is repressed by Hsp90. The depletion of Hsp90 induces Hsf1 phosphorylation and upregulates Hsf1 targets, and depletion of *HSP90* activates *HSF1* (Zou et al., 1998; Diezmann et al., 2012; Leach et al., 2012a; Leach et al., 2016; Kijima et al., 2018; Veri et al., 2018). Our results showed that curcumin induced the transcription of *HSF1* in the wildtype strain, the same as in the Dox-mediated *tetO-HSP90/hsp90Δ* strain (**Figures 5A,B**). These experiments suggested that curcumin reduces *HSP90* expression directly, and not dependently on Ahr1 and Hsf1. Interestingly, we thought that *HSP90* expression in the *tetO-HSP90/hsp90Δ* strain would not be influenced by curcumin, because the *HSP90* promoter was replaced by the *tetO* element. However, curcumin reduced *HSP90* mRNA in the *tetO-HSP90/Δhsp90* strain (**Figure 4C**). Hence, we assumed that the induction of *HSP90* mRNA degradation occurred in the presence of curcumin. After actinomycin D inhibited transcription in the cells, the *HSP90* mRNA amount was measured in the presence or absence of curcumin (**Figure 5C**). Although *HSP90* mRNA was degraded gradually after inhibition of mRNA synthesis, curcumin accelerated its degradation (**Figure 5D**). In contrast, faster degradation of *ACT1* mRNA was not observed when adding curcumin (**Figure 5D**). Curcumin has been reported to change DNA methylation in human cancer cells as an epigenetic modification (Link et al., 2013). This indicates that curcumin might have inhibited *HSP90* expression at the post-transcriptional level by DNA methylation changes.

ABC transporters, including Cdr1 and Cdr2, are drug efflux pumps that play an important role in the development of multidrug resistance in *C. albicans*. Previous studies have shown that curcumin inhibits ABC transporters, including *C. albicans* Cdr1, Cdr2, and *Saccharomyces cerevisiae* Pdr5p, competitively (Sharma et al., 2009; Sharma and Prasad, 2011). In addition, recent studies (Diezmann et al., 2012; Leach et al., 2012a) have shown that a reduction of *HSP90* reduces the protein level of Cdr1 in *C. albicans*. In this study, curcumin downregulated *CDR1* gene expression, suggesting that the reduction of *HSP90* expression by curcumin led to a decrease in *CDR1* expression.

Previous study showed that curcumin was able to modulate multidrug resistance (MDR) phenotype of *C. albicans* (Garcia-Gomes et al., 2012). In this study, the Nile red accumulation assay was used to analyze the efflux pump activity in *C. albicans*. Nile red is a known substrate of ABC-transporters Cdr1, Cdr2 and Mdr1 in *C. albicans* (Ivnitski-Steele et al., 2009), the cells can efflux Nile red immediately. Our data showed that curcumin drastically decreased the efflux pump activity of the wildtype strain after a 2-h incubation with curcumin (**Figures 7B, C**). This was due to the reduction of *CDR1* expression by curcumin. The depletion of *HSP90* in the *tetO-HSP90/Δhsp90* strain also led to a decrease in efflux pump activity, while *HSP90* overexpression maintained the efflux pump activity of the *P_ADH1_-HSP90* strain in the presence of curcumin (**Figures 7D-G**). Curcumin is also known to be an inhibitor of Cdr1 activity (Pearson et al., 1999; Falagas et al., 2006; Piddock, 2006; Sharom, 2008; Wu et al., 2011). In this study, without affecting gene expression of ABC-transporters, curcumin also blocked efflux pump activity for Nile red in the wild type (**Figures 9**, **10**). This finding showed that curcumin inhibited the activity of efflux pumps such as Cdr1, while low efflux pump activity remained because Cdr2 and Mdr1 were not inhibited by curcumin.

**Figure 11:**
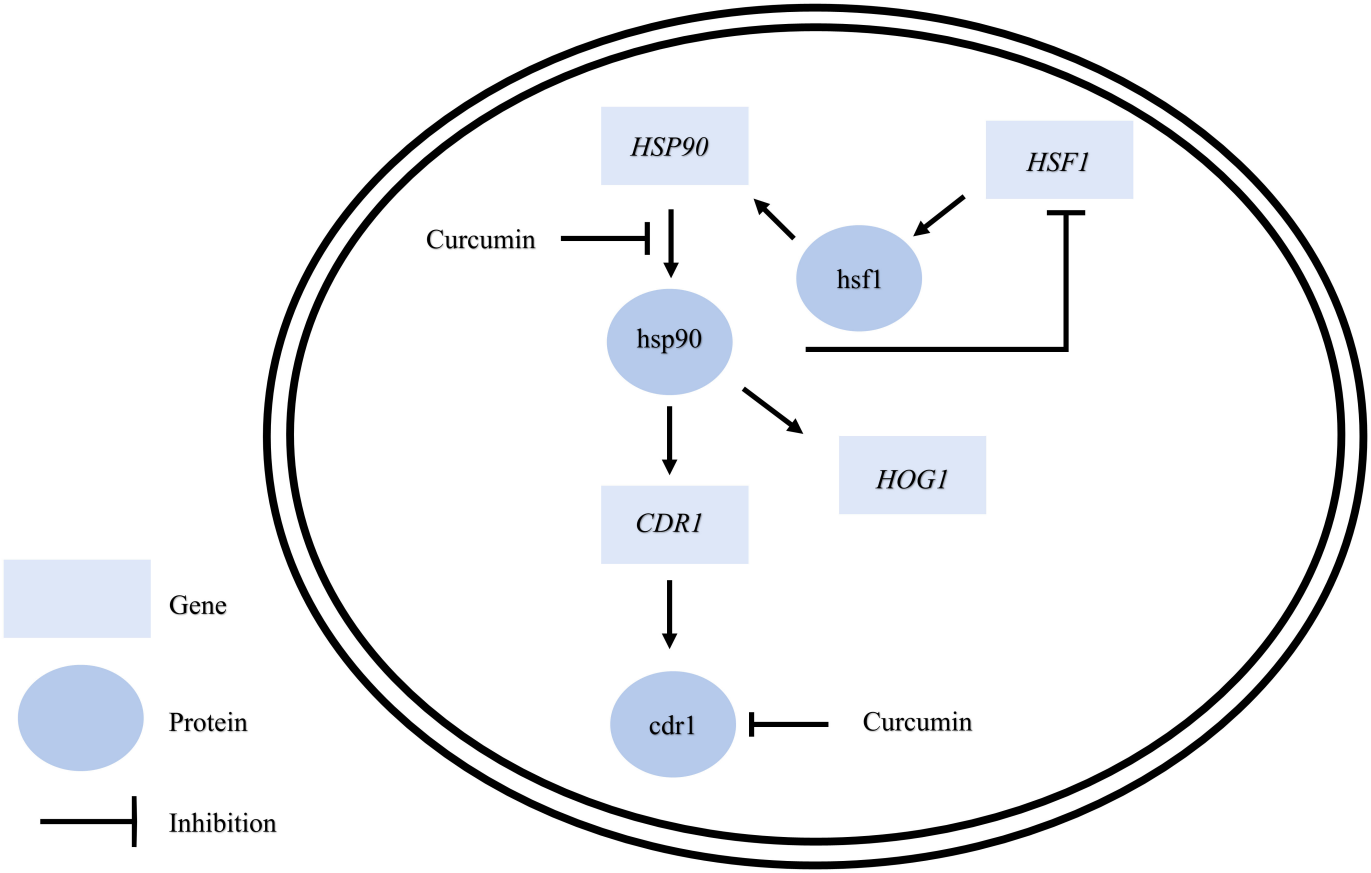
Model for the mechanism of curcumin’s effects on *C. albicans*.

Taken together, this study sheds new light on the functions of curcumin (**Figure 11**). Curcumin affects not only drug efflux pumps such as Cdr1 but also *HSP90* expression, mainly at the post-transcriptional level. Hence, the natural product curcumin and its derivatives may be used as antifungals to inhibit drug efflux pumps and cell growth of *C. albicans*. However, the complex mechanism by which curcumin affects *C. albicans* needs to be further explored.

## Data availability statement

The original contributions presented in the study are included in the article/**Supplementary Material**. Further inquiries can be directed to the corresponding author.

## Author contributions

YL and SK conceived the study. YL performed the experiments; YL, TW, and XC collected and analyzed the data. KO and HS gave support YL, XC and SK wrote the manuscript. All authors contributed to the article and approved the submitted version.

## Funding

We received internal research funds from Tokyo Institute of Technology.

## Acknowledgments

We thank the Open Research Facilities for Life Science and Technology in the Tokyo Institute of Technology for technical support and equipment.

## Conflict of interest

The authors declare that the research was conducted in the absence of any commercial or financial relationships that could be construed as a potential conflict of interest.

## Publisher’s note

All claims expressed in this article are solely those of the authors and do not necessarily represent those of their affiliated organizations, or those of the publisher, the editors and the reviewers. Any product that may be evaluated in this article, or claim that may be made by its manufacturer, is not guaranteed or endorsed by the publisher.
