# Supplementary figures and images for: Curcumin affects function of Hsp90 and drug efflux pump of Candida albicans
Source: Front Cell Infect Microbiol. 2022 Sep 27;12:944611. doi: 10.3389/fcimb.2022.944611 (PMC9551236; doi:10.3389/fcimb.2022.944611)

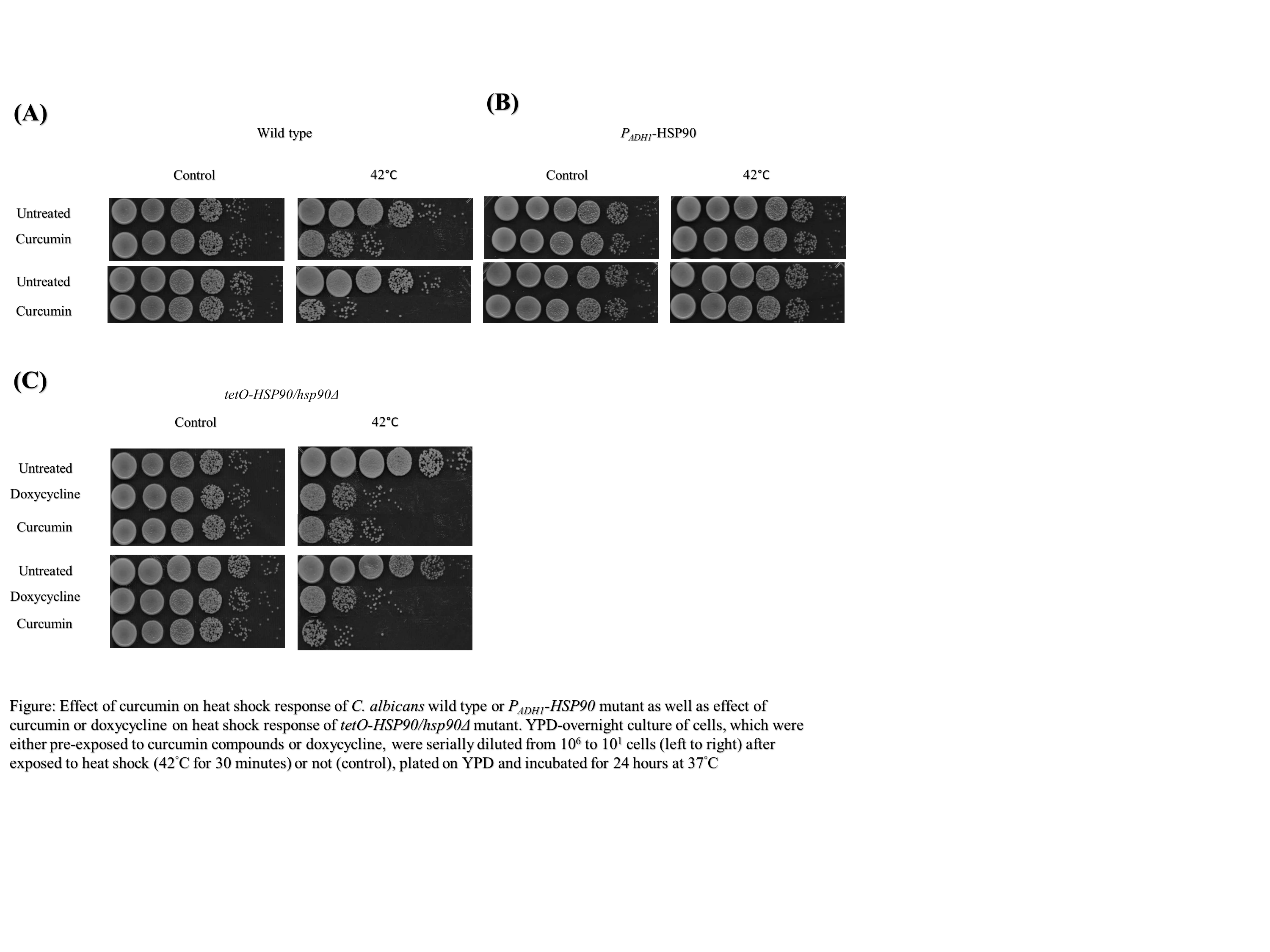

Supplement: Supplementary file 1 [file Image_1.tif]

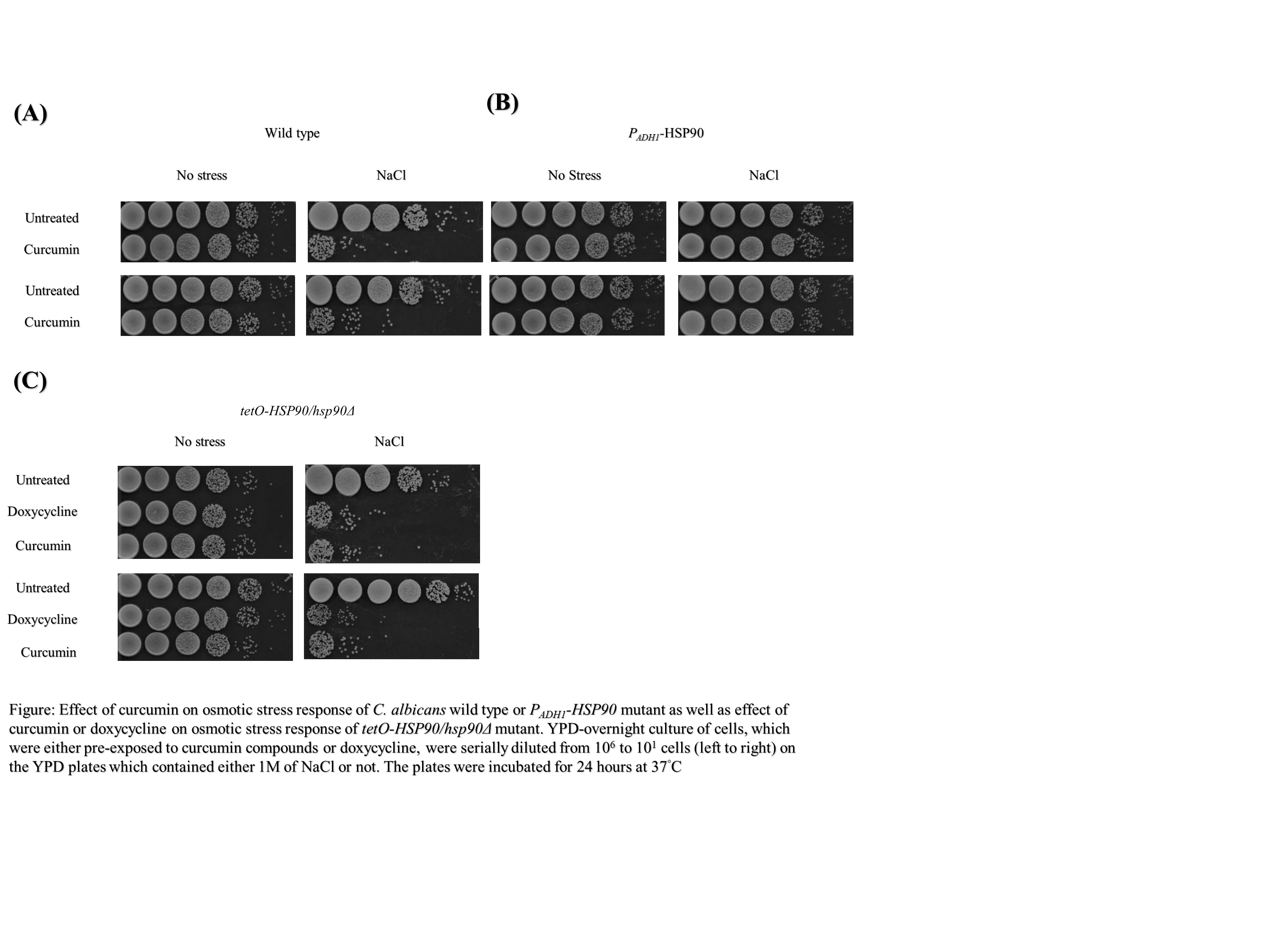

Supplement: Supplementary file 2 [file Image_2.tif]
